# Brand Love: Role of Online Customer Experience, Value Co-creation, and Relationship Quality

**DOI:** 10.3389/fpsyg.2022.897933

**Published:** 2022-07-15

**Authors:** Khurram Mustafa, Farooq Ahmad, Muhammad Nawaz Qaisar, Shagufta Zada, Saqib Jamil, Naveed Anwer, Kausar Fiaz Khawaja, Alejandro Vega-Muñoz, Nicolás Contreras-Barraza, Syed Ali Raza Hamid, Shahida Mariam

**Affiliations:** ^1^Faculty of Management Sciences, University of Okara, Okara, Pakistan; ^2^Department of Business Administration, Fatima Jinnah Women University, Rawalpindi, Pakistan; ^3^National Accountability Bureau, Peshawar, Pakistan; ^4^Faculty of Management Sciences, National University of Modern Languages, Islamabad, Pakistan; ^5^Business School, Henan University, Kaifeng, China; ^6^Department of Business Administration, ILMA University, Karachi, Pakistan; ^7^Faculty of Management Sciences, Shaheed Zulfikar Ali Bhutto Institute of Science and Technology, Larkana, Pakistan; ^8^Lahore Business School, University of Lahore, Lahore, Pakistan; ^9^Faculty of Management Sciences, International Islamic University, Islamabad, Pakistan; ^10^Public Policy Observatory, Universidad Autónoma de Chile, Santiago, Chile; ^11^Facultad de Economía y Negocios, Universidad Andres Bello, Viña del Mar, Chile; ^12^Hamdard Institute of Management Sciences, Hamdard University, Islamabad, Pakistan; ^13^Government Associate College, Rawalpindi, Pakistan

**Keywords:** online customer experience, relationship quality, brand love, value co-creation, multi-attribute utility theory, the attitude-behavior-context theory

## Abstract

Customer experience is a source of retailers’ long-term competitive advantage. This study has examined the relationship between online customer experience and brand love through the mechanism of relationship quality in the context of online shopping in Pakistan. The moderating effect of value co-creation on the relationship of online customer experience with relationship quality and brand love has also been examined. Data were collected from a purposive sampling of 189 online customers in an online survey. Results showed that online customer experience significantly impacts customer relationship quality, which leads to brand love. The relationship between online customer experience and relationship quality is found more robust at high levels of value co-creation. However, we observed a significant negative moderating effect of value co-creation on the direct relationship between online customer experience and brand love. It suggests that including customers in the value co-creation process and affording them a pleasurable online shopping experience may be an excellent way to enhance customer relationship quality and brand love. Theoretical and practical implications of these findings are discussed.

## Introduction

Customers are increasingly turning to the internet to fulfill their buying needs. International dynamics, sophisticated infrastructure, consumer lifestyles, and the mushroom expansion of information communication and technology drive this behavior. These reasons have heightened consumers’ demands regarding current trends and increased living conditions that are socially acceptable. The most valuable part of online shopping is enhanced customer experience through product and service comparisons (Sivanesan, 2017). Customers of various ages, particularly the youth, have demonstrated a strong presence on numerous online buying platforms, dramatically expanding their buying possibilities and choices (Ellison et al., 2021).

A firm’s brand is its intangible asset (Paul, 2018). Recent branding advancements are centered on investigating the emotional component of branding. Literature also suggests that, in addition to attitudinal factors, consumers’ behavior is influenced by their feelings about a brand (Sniehotta et al., 2014). As a result, these feelings play a crucial part in determining the brand’s fate. As a result, consumer-brand connections have taken center stage in the branding literature. They have a significant part in developing a brand (Wang et al., 2018). The rise of traditional conceptions such as brand love and relationship quality has offered crucial inputs to marketers for placing their brands on social media utilizing various techniques. They have recently gotten a lot of notice (Joshi and Garg, 2021). Because today’s customers rely on brands to enhance their personalities and see brands as representatives of their inner selves, the value of a brand grows.

The triangular theory of love proposed by Steinberg (1986) gave rise to brand love—a psychological construct. It is the degree of a happy customer’s deep emotional commitment to a specific brand (Carroll and Ahuvia, 2006). It takes a lot of effort on the marketer’s side to maintain a customer’s deep connection with the brand. A customer’s enthusiasm for a brand depends on its quality and endurance. It necessitates a comprehensive effort in brand marketing, consumer experience, internet presence, continual brand enhancement, and customer relationship management. On the other hand, brand love may lead to higher-order feelings like loyalty, good word of mouth (WOM), active participation, and readiness to pay a premium for a particular brand (Joshi and Garg, 2021).

As an overall concept, relationship quality comprises satisfaction, trust, and commitment (Lemon and Verhoef, 2016). A customer’s overall assessment of the strength of their relationship with a company is characterized as relationship quality (Palmatier and Grewal, 2006). As previously stated, customer satisfaction is linked to their overall assessment of its products (Anderson et al., 1994). Customer propensity to rely on an exchange partner with whom a particular level of confidence has been created is how trust is defined (Moorman et al., 1993). Customer commitment to the company requires trust, which is why trust and commitment are at the heart of the relationship marketing architecture. Partner reciprocity and non-opportunistic behavior are two factors that contribute to relationship trust (Vivek et al., 2012). A customer’s enduring desire to sustain a valued connection (Moorman et al., 1993) is characterized by commitment, and it reveals the nature of the relationship (Palmatier et al., 2007). The literature lacks its connection with brand love and online customer experience. Hence, this research aimed to fill that gap.

## Theory and Hypotheses Development

Discovering the implicit meaning of love that customers use when they say they love a particular brand or product is the first step in understanding brand love. A prototype is a collection of characteristics (i.e., prototype traits) that people connect with a specific sort of object, in this case, love (Fehr, 1988). These characteristics are categorized into a core or typical exemplar of that category, such as love, or a subclass, romantic love, parental love, and brand love (Shaver et al., 1987). Customers often open their remarks regarding beloved brands with a list of their impressions of the brand’s many appealing attributes, such as excellent performance, trustworthiness, and attractive design. Instead, adored brands were lauded for being the finest available (e.g., most significant in every manner, best value for money, and top on some crucial feature), and simply knowing that a better brand existed was frequently cited as a cause for not appreciating a specific brand (Batra et al., 2012). Albert et al. (2009) proposed that brand love is similar to interpersonal love. The phrase “brand love” was coined by Carroll and Ahuvia (2006), who demarcated it as a revolutionary brand sentimental predisposition. According to Bergkvist and Bech-Larsen (2010), consumers organize the sum of cognition, emotions, and behaviors in a mental prototype because they adore a particular brand.

In contrast, according to Batra et al. (2012), brand love is the sum of cognition, emotions, and behaviors that consumers organize in a mental prototype. After all, they adore a particular brand. Respondents used positive emotional adjectives to describe their experiences with liked brands, and this tendency was significantly stronger for loved consumption behaviors. This effect encompassed the lower-arousal emotions of affection (Thomson et al., 2005) as well as warm-hearted feelings (Richins, 1994) that are characteristic of companionate love (Hatfield et al., 2012).

**Figure 1:**
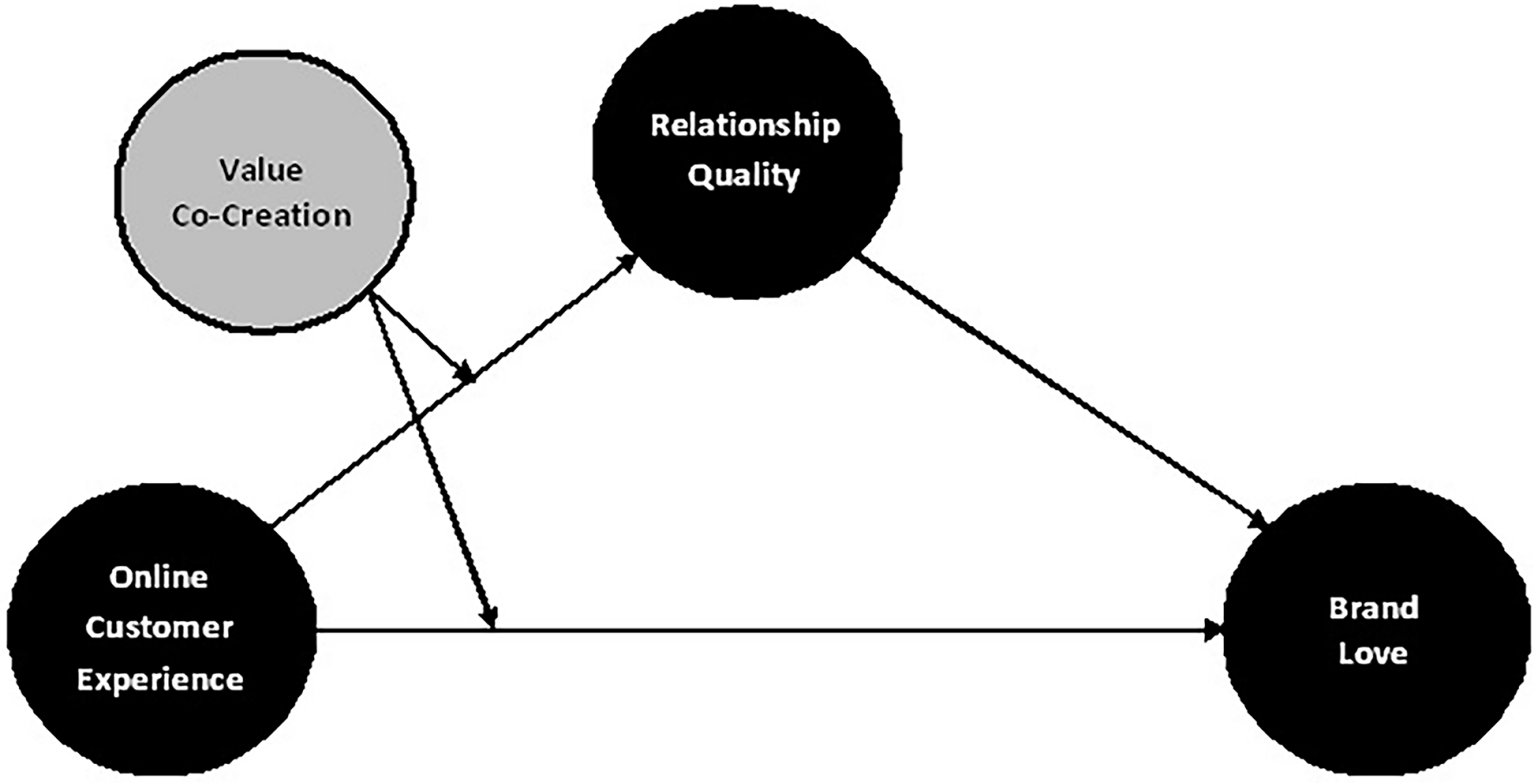
Proposed research model.

The nature of the products plays a significant role in developing and improving online shopping. As a result, merchants must be aware of the factors influencing customers’ opinions and the implications, such as buy intents and online store loyalty. Researchers used the multi-attribute utility theory (MAUT) and the attitude-behavior-context theory (ABC) to analyze the proposed phenomenon. MAUT assists in the decision-making process. It is employed in this study because it provides a method for systematically assessing and weighing different choices. It helps decision-makers access and choose from possibilities (Geoffrion et al., 1972). It improves the decision-making process by offering a framework for finding enhanced attributes across all key performance indicators (Collins et al., 2006). In many scenarios, the ABC theory postulated environmental and consumer behavior. The concept was proposed by Guagnano et al. (1995), and it illustrates how contextual factors can help predict consumer attitudes about showing specific actions. Simply having a positive outlook, according to the researchers, is insufficient to legitimize consumer behavior in online shopping (Goh and Balaji, 2016). It is argued that the association between attitude and behavior is more robust when structural factors stimulate behavior at a moderate level rather than when it is extended to the point where even those with the most negative attitudes engage in it (Olander and Thogersen, 2005). This study has assessed the impact of online customer experience on brand love *via* relationship quality using value co-creation (see Figure 1).

### Online Customer Experience

A customer’s impression after interacting with products, services, and businesses and establishing a perception based on sensory data is referred to as an online customer experience (Anshu et al., 2022). It is crucial to deliver customer satisfaction, create expectations, gain trust and confidence, retain loyal customers, and form effective bonds (Slack and Singh, 2020). Experience is a broad term used in various topics and learning environments. Academics and professionals are interested in the subject; however, researchers argue that research is scarce and fragmented (Bilgihan et al., 2016). According to Hult et al. (2019), customer experience is an internal and subjective reaction between a customer and an enterprise during direct and indirect contact. It encompasses various aspects of service quality provided by the company, including advertising, packaging, function, user-friendliness, and product and service reliability. As a result, access to a widely acknowledged study on online consumer experiences in retail is limited (Petermans et al., 2013). Research may be divided into four categories: product experience, service procedure, shopping environment, and staff-service experience (Pei et al., 2020).

### Online Customer Experience and Brand Love

Brand love is outlined as a satisfied consumer’s emotional attachment to a specific brand, which can be established when the consumer is proficient in (a) generating identity and supporting and conveying a self-identity, (b) activating his or her memory, and (c) inspiring his or her delight and exuberance sentiment (Albert et al., 2009; Batra et al., 2012). The concurrent self-presentation feature of technology, for example, allows e-shoppers to edit their self-representation and adorn the stature of their avatar or online self to their heart’s content in the product usage simulation (Hanus and Fox, 2015; Huang and Liao, 2015). Compared to ordinary Web page browsing, an e-retailing environment is more interested in creating a shopping setting closer to the consumer’s self-concept and self-identity. According to a consumer psychology study, individuals who can fully display an individual self-image will be fit to develop, shape, and create close brand associations such as brand love (Chang, 2012). The second capacity, memories activation, leads to a new brand inducing nostalgic feelings in customers by encouraging people to recall past pleasant memories of themselves and connecting new brand information with memories and experiences (Albert et al., 2008, 2009). Compared to standard webpage surfing, the many enhanced sensory inputs online offer realism and a dynamic experience to e-shoppers. An e-retail environment could produce a higher first-person effect in simulating products (Roy et al., 2017). Escalas (2004) experimental results demonstrated that first-person product usage simulations are the most effective in evoking a consumer’s memory and integrating this memory with new brand information. As a result, the e-retail environment is better suited to a brand activation setting.

Brand love is a growing yet vital idea in gaining a deeper understanding of online consumer-brand relationships (Huber et al., 2015). Carroll and Ahuvia (2006) demarcated brand love as a pleased customer’s robust and emotional commitment to a particular brand name. As marketers’ focus turns from pushing distinctive online selling propositions to creating emotional relationships with customers, the value of a notion like brand love will only grow in modern marketing. A favorable brand attitude, brand trust, self-expression, pleasant customer experience, a sensation of psychological attachment to the brand in an online way, and a brand’s hedonic worth are all antecedents of brand love, according to existing research (Batra et al., 2012; Albert and Merunka, 2013; Sarkar and Sarkar, 2016). However, more study is needed to discover and confirm these antecedents (Albert and Merunka, 2013; Fetscherin, 2014). This study will provide some insights into this literature gap. If a brand leads in technology, consumers love it; for example, Samsung is appreciated because of its reputation as a technology leader (Trivedi, 2019). Brand love is an essential notion of noticing compared to brand attitude or satisfaction since it displays a more robust form of online consumer–brand interaction (Karjaluoto et al., 2016). MAUT explains the proposed link in a way that customers having a positive online shopping experience would be in a better position to decide whether to buy the desired products from an online forum or not. It also explains that ease in decision-making for online shopping originating from a positive online experience would lead to long-term brand love. This study aims to see if the online customer experience leads to love for the brand.

*H1*: A positive online customer experience has a significant positive impact on brand love.

### Mediating Role of Relationship Quality

Web 2.0’s interactivity allows consumers to actively participate in marketing activities to strengthen the online consumer-brand relationship (Sawhney et al., 2005; Hennig-Thurau et al., 2010; Rappaport, 2010). Consumers can use the website to find product-related information quickly and easily and actively govern the search and usage of information (Bezjian-Avery et al., 1998; Hennig-Thurau et al., 2010). By monitoring and capturing open, spontaneous, and up-to-date customer insights about the brand, the development of online communication helps the brand create a close relationship with consumers (Yoon and Youn, 2016). According to a large body of work on relationship marketing, online communication is an excellent strategy for building consumer-brand relationships and increasing consumer satisfaction (Thorbjørnsen et al., 2002; Yoon et al., 2008; Hennig-Thurau et al., 2010). Brand experience is a set of brand-consumer interactions elicited by various brand-related stimuli (Brakus et al., 2009). These conversations can take place in person or online. If consumers have a pleasant and distinctive online experience with a brand, they are more likely to trust it, have a satisfying connection, and be committed to it. In other words, a positive relationship with customers is a crucial outcome of the brand experience (Morgan-Thomas and Veloutsou, 2013) through the internet. As a result, this study predicts relationship quality as a positive mediating relationship between online customer experience and brand love.

Recent research shows that the importance of online brand experience in the relationship-building process is supported by evidence (Iglesias et al., 2011). First, a study by Brakus et al. (2009) to establish the brand experience scale demonstrated a link between brand personality and two behavioral outcomes: consumer satisfaction and loyalty. It is in line with the previous research (Yoon and Youn, 2016), which showed that the emotive and behavioral components of online brand experience were positively related to commitment and trust. Iglesias et al. (2011) found that excellent brand experience is indirectly affected. They emphasized forming effective relationships with customers in building a long-term brand relationship. It is worth noting that most previous customer experience research has focused on the repercussions of customer experience in an offline setting (Yoon and Youn, 2016). Beyond this line of inquiry, the current study adds to the understanding of online customer experience by examining it in the context of an online marketing environment and examining its role between perceived relationship quality and brand love. Yoon et al. (2008) looked at how online interaction influences consumers’ “perceptions of e-retailers” efforts to develop relationships, affecting relationship quality.

Similarly, the current study predicts that the perceived online interaction influences brand experience on the site, affecting relationship quality. This hypothesis argues that relationship quality indirectly affects brand love *via* online customer experience. This study needs to find a good association between customer experience on the website and relationship quality to validate the role of brand love. MAUT explains the proposed link in a way that customers having a positive online shopping experience would be in a better position to decide whether to buy the desired products from an online forum or not. It will create a sense of the excellent relationship quality and promote long-term brand love. Accordingly, we predicted that:

*H2*: Relationship quality mediates the association between online customer experience and brand love.

### Moderating Role of Value Co-creation

According to previous research, co-creation is commonly viewed as a process of interaction with or influencing the stakeholders, and brands constantly evolve among various actors (Sonja et al., 2022). The concept of value co-creation is based on the idea that consumers are co-creators of value and that businesses can only propose value propositions rather than produce value (Vargo and Lusch, 2004, 2008). This trend in online branding has piqued the interest of academics. Previous value co-creation research has been focused on offline interactions and encounters between a firm and a visitor (Payne et al., 2009). The situation has recently shifted, and virtual network dialogues are being studied (Ramaswamy, 2009; Hoyer et al., 2010). The main focus is on interactions between providers and customers outside the providers’ sphere of influence (Grönroos and Voima, 2013). Despite significant advancements in marketing research, value co-creation in online customer experience has taken on a too restricted perspective. This exploration looks to grow the extent of concentration by remembering business-to-client communications for the value co-creation model and moving from application to offline to online brands. As per studies, value co-creation enhances the effect of encounters, working on apparent worth, uplifting outlook (Meng and Cui, 2020), buying aim, and conduct (Pee, 2016). Research indicates that value co-creation moderating affects the results of experiences (Meng and Cui, 2020). Clients return to online retailers who like their thoughts, which has brought about internet-based client traffic being coordinated to organizations where their thoughts are looked for, assessed, and included, bringing about value co-creation (Pathak et al., 2017).

Brand love, unlike interpersonal love, is a condition in which reciprocity is not expected because brands are not expected to express emotions (Batra et al., 2012). People do not frequently name their emotions toward brands as “love.” The brand love construct better define a relationship between consumers and a brand rather than an episodic emotion in online consumer-brand relationships. Carroll and Ahuvia (2006) defined brand love as the degree of passion and emotional attachment a satisfied consumer has for a particular trade name.

Passion and emotional attachment are two of the critical characteristics of brand love. Passion has been linked to brand love (Albert et al., 2008) and emotional attachment (Batra et al., 2012). A strong desire for a brand reflects higher-order emotions (Thomson et al., 2005; Batra et al., 2012). On the other hand, the emotional connection is characterized as an emotion-laden target-specific bond between a person and a particular product, similar to brand love. Researchers agree that attachment is an essential component of brand love (Thomson et al., 2005; Carroll and Ahuvia, 2006; Albert et al., 2008; Batra et al., 2012; Loureiro et al., 2012). According to Batra et al. (2012), brand attachment is an emotional bonding with and connection to the brand that may cause separation distress. This study addresses a gap in the literature by evaluating the moderating impact of value co-creation on online customer experience and brand love. MAUT explains that customers having a positive online shopping experience would develop a better relationship quality and brand love. The ABC further predicts that contextual and behavioral factors of value co-creation would strengthen the effect of positive online customer experience to build strong relationship quality and brand love. Thus, we expected that:

*H3*: Value co-creation moderates the association between online customer experience and relationship quality, such that this relationship is stronger at a high level of value co-creation.

*H4*: Value co-creation moderates the association between online customer experience and brand love, such that this relationship is stronger at a high level of value co-creation.

## Materials and Methods

### Pilot Study

A pilot study was conducted with participants who made purchases over the internet. The pilot study enlisted the participation of 50 young adults. They were asked to talk about their experiences with online stores and brands. Customers said they shop online through social media platforms like Facebook and Instagram. They also talked about the brands they bought from online retailers. For example, they purchased outfits, footwear, jewelry, bracelets, cellphones, and other fashion items. The pilot study results determined what consumers want and buy from online retailers. As a result, it set the stage for this research and outlined the process.

### Sample and Technique

Data was collected from online customers in Pakistan in a cross-sectional survey using a structured online questionnaire. Most of the participants from different social strata were students at different universities. Younger customers were preferred because they are more likely to be loyal to online retailers in the long run and are more willing to try new things. Only online shopping participants were eligible to participate in the online poll distributed across several online platforms during 15th January and 14th February 2022. For this study, a total of 189 valid responses were used. Table 1 shows the demographic information. The statistical programs SPSS and Smart-PLS were used to analyze the data and draw conclusions. The purposive sampling (non-probability sampling) method selected participants based on their age limit and online shopping experience. They had to be between the ages of 16 and 35, and they had to have done some online shopping at least once. This strategy was also used as the opening question in a survey. Participants who did not meet the criteria were not allowed to participate in the survey. It is how the data from the target sample was gathered.

**Table 1:** Participants’ demographic details.

Characteristics	Participants (*N* = 189)	
	Frequency	Percentage
**Gender**		
Male	137	72.5
Female	52	27.5
**Age**		
16–20 years	69	36.5
21–25 years	98	51.9
26–30 years	10	5.3
31–35 years	12	6.3
**Educational qualification**		
Doctoral	9	4.8
Masters	25	13.2
Graduation	106	56.1
Intermediate	49	25.9
**Occupation**		
Student	162	85.7
Business	2	1.1
Service	14	7.4
Self-employed	6	3.2
Housewife	1	0.5
Other	4	2
**Monthly household income**		
Less than Rs. 25,000	71	35.6
Rs. 25,000 to Rs. 49,999	68	36
Rs. 50,000 to Rs. 74,999	21	11.1
Rs. 75,000 to Rs. 99,999	13	6.9
Rs. 100,000 or more	16	8.5
**Time duration since buying products online**		
Less than 1 month	67	35.4
1–6 months	50	26.5
6–12 months	25	13.2
More than 1 year	47	24.9
**Online products purchase frequency**		
Daily	2	1.1
Once a week	19	10.1
Fortnightly	6	3.2
Monthly	67	35.4
Rarely	95	50.3
**Value of the online shopping (rupees per purchase)**		
Rs. 1,000 or less	62	32.8
Rs. 1,001 to Rs. 2,000	53	28
Rs. 2,001 to Rs. 3,000	36	19
Rs. 3,001 to Rs. 4,000	13	6.9
Rs. 4,001 to Rs. 5,000	10	5.3
Rs. 5,001 or more	15	7.9

### Measures

This study used a seven-point Likert scale ranging from 1 (strongly disagree) to 7 (strongly agree) to collect data from study respondents. In addition, the scales and sample items of study variables are listed below.

#### Online Customer Experience

Product experience, shopping environment, staff service experience, and shopping procedure were the four dimensions of online customer experience used in this study. All four dimensions of online customer experience and their scales were adapted from Parasuraman et al. (1988). Product experience (PE, α = 0.74) contains five items, such as the item is “This online store has a variety of categories and colors. “The shopping environment (SE, α = 0.79) comprises three items; one of the items is “This online store shows neat and attractive web design.” Staff service experience (SSE, α = 0.81) has three items: “The staff of this online store shows frequent communication with the customer and good service attitude.” At last, the shopping procedure (SP, α = 0.77) has three items, for example, “This online store ensures the availability of pictures and reviews.” All four measures of online customer experience indicated good reliability in this study (PE: CR = 0.90, α = 0.87; SE: CR = 0.89, α = 0.85; SSE: CR = 0.94, α = 0.92; and SP: CR = 0.89, α = 0.85).

#### Value Co-creation

The scale of value co-creation is adapted from Prahalad and Ramaswamy (2004) in the form of six items (α = 0.63), for example, “I am actively involved when this online store develops new solutions for me.” The measure of value co-creation exhibited good reliability (CR = 0.95, α = 0.93).

#### Relationship Quality

A total of 10 items of relationship quality have been adapted as four items for the brand trust (Gurviez and Korchia, 2003), three items for brand commitment (Fullerton, 2003), and three items for brand satisfaction (Aaker et al., 2004). One item of the brand trust is “I trust the product quality of this online brand.” The item for brand commitment is “I feel emotionally attached to this online brand.” An item representing brand satisfaction is “I am satisfied with this online brand’s products.” The measure of value co-creation exhibited good reliability (CR = 0.95, α = 0.93).

#### Brand Love

There are 13 items of brand love adapted from Shimp and Madden (1988), for example, “I feel much affection for this online brand.” The measure of value co-creation exhibited good reliability (CR = 0.95, α = 0.93).

## Results

### Measurement Model

**Figure 2:**
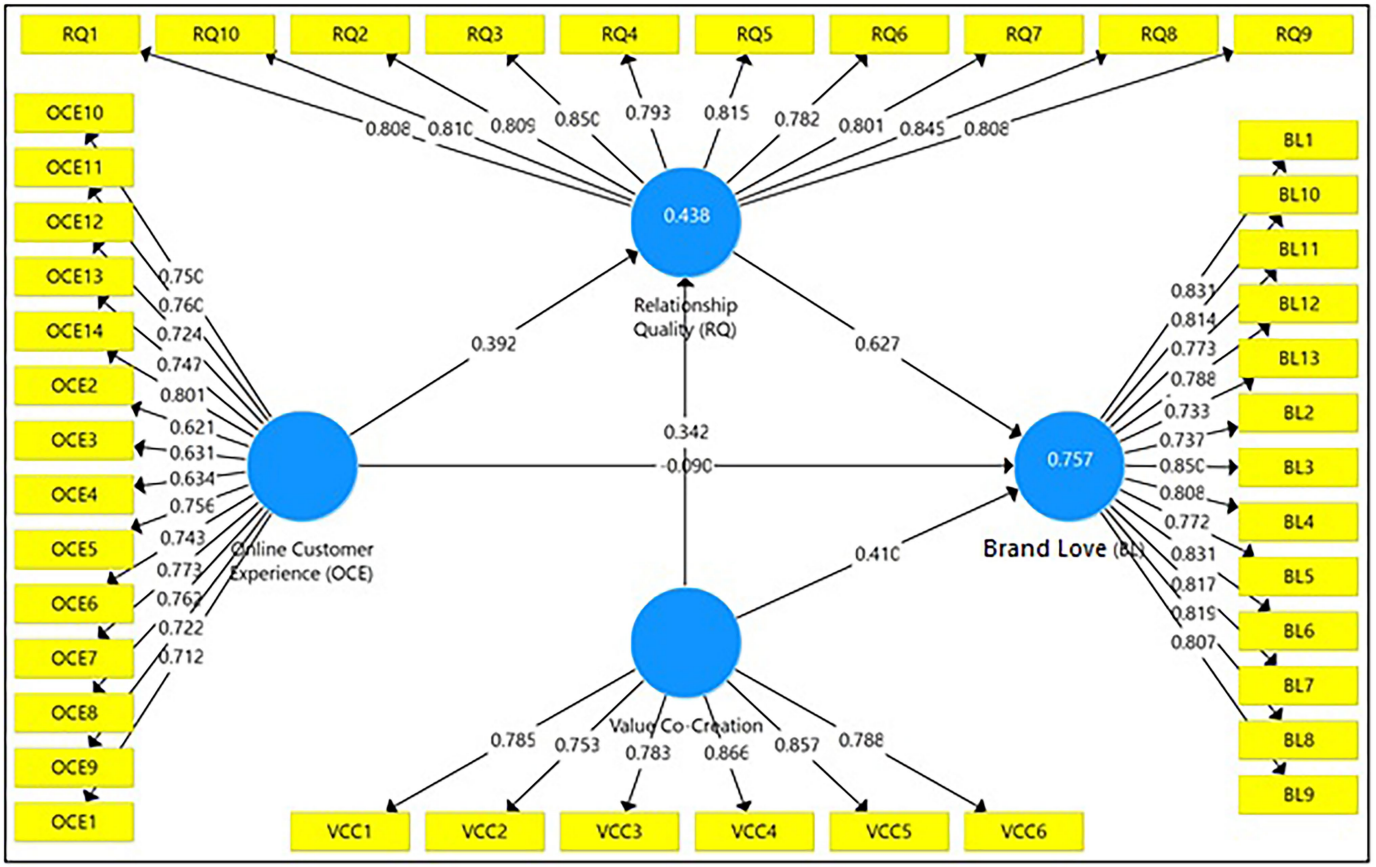
Measurement model.

The measurement model used in this study showed a high construct validity and reliability level. Except for one item for online customer experience (OCE = 0.562), almost all factor loadings surpassed the benchmark of 0.700 in Figure 2. Cronbach’s Alpha (>0.700), composite reliability (>0.700), and average variance extracted (AVE) values over 0.500 fulfilled the minimal standards, as shown in Table 2. All constructions’ composite reliabilities were likewise higher than their respective AVEs. These results also looked at discriminant validity, which requires that the square root of all AVEs be greater than the correlations between constructs (Fornell and Larcker, 1981). Table 2 reveals that square roots of AVEs are larger than inter-construct correlations except for OCE, indicating that discriminant validity has been established. As a result, the structural model’s hypothesis testing assumed that the measurement model was sufficiently trustworthy and valid.

**Table 2:** Construct reliability and validity.

	**α**	CR	AVE
Online customer experience	0.930	0.940	0.527
Relationship quality	0.943	0.951	0.660
Value co-creation	0.933	0.947	0.749
Brand love	0.946	0.952	0.605

α, Cronbach’s Alpha; CR, Composite reliability; AVE, Average variance extracted.

### Descriptive and Correlational Analyses

The mean, standard deviations, and correlation coefficients for the research variables are shown in Table 3. As expected, online customer experience indicated significant positive correlations with relationship quality (*β* = 0.607, *p* < 0.01) and brand love (*β* = 0.349, *p* < 0.01). Value co-creation reflected a significant positive correlation between relationship quality (*β* = 0.649, *p* < 0.01) and brand love (*β* = 0.371, *p* < 0.01). In this investigation, these correlations revealed the possibility of forecasting theoretical connections without the danger of multicollinearity.

**Table 3:** Correlations and discriminant validity.

	Mean	SD	1	2	3	4
1. Online customer experience	5.504	1.067	**0.726**			
2. Relationship quality	5.370	1.229	0.607^**^	**0.812**		
3. Value co-creation	4.980	1.371	0.402^**^	0.649^**^	**0.866**	
4. Brand love	4.904	1.198	0.349^**^	0.606^**^	0.371^**^	**0.778**

*N* = 189. SD, Standard deviation. Bold diagonal values are √AVE.

** *p* < 0.01.

### Structural Model and Hypothesis Testing

**Figure 3:**
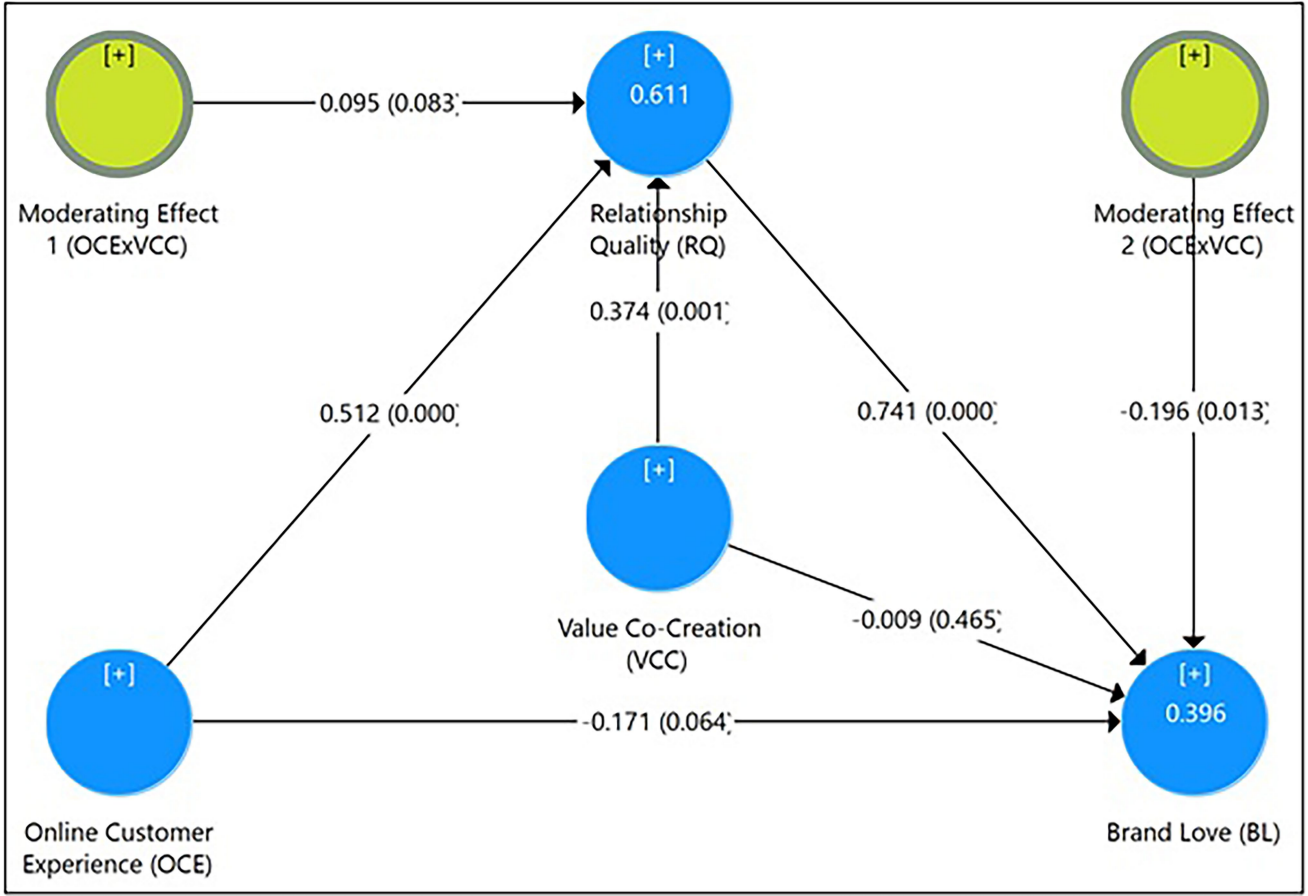
Estimated path model.

The path analysis was done using a partial least square (PLS) method. The direct, indirect, and total effects were studied to evaluate the proposed hypotheses, as indicated in Table 4. We looked at the direct and cumulative effects of online customer experience (OCE) on brand love (BL) and relationship quality (RQ). Second, the direct and total effects of OCE on RQ and the effects of RQ on BL were investigated. Finally, the four-step approach (Baron and Kenny, 1986) was used to observe the mediation impact of RQ on the link between OCE and BL. Finally, the analysis examined the moderating effects of VCC on RQ and BL, respectively. The complete moderated-mediation model and the estimated PLS path model (Figure 3) show that OCE (*β* = 0.512) and VCC (*β* = 0.374) and their interaction term (OCE × VCC; *β* = 0.095) explain a 61.1% variance in RQ (R^2^ = 0.611).

**Table 4:** Path analysis.

Path	Effect (*t*-value)	Hypotheses	Outcome
OCE **→** BL	0.209^*^(1.944)	H_1_	Supported
OCE **→** RQ **→** BL	0.379^**^(4.515)	H_2_	Supported
VCC **→** RQ	0.374^**^(3.081)	−	−
VCC **→** RQ **→** BL	0.277^**^(2.915)	−	−
Moderating Effect 1: OCExVCC **→** RQ	0.095^*^(1.387)	H_3_	Supported
Moderating Effect 2: OCExVCC **→** BL	−0.196^**^(2.213)	H_4_	Supported

BL, Brand love; OCE, Online customer experience; RQ, Relationship quality; VCC, Value co-creation. *p < 0.05; **p < 0.01.

According to Hypothesis 1, OCE has a substantial positive relationship with BL. The direct effect (*β* = 0.209, *t* = 1.944, *p* < 0.05) validates the hypothesis (see Table 4). According to Hypothesis 2, RQ mediates the link between OCE and BL. The stated path analysis (see Table 4) indicated that OCE has an indirect influence on BL (*β* = 0.379, *t* = 4.515, p < 0.01) through RQ. As a result, hypothesis 2 was found to be true. VCC positively moderated the associations between OCE and RQ, according to hypothesis 3. The results of the path analysis revealed that VCC made a significant contribution to RQ (*β* = 0.374, *t* = 3.081, *p* < 0.01) and its moderating effect (OCE × VCC) on RQ (*β* = 0.095, *t* = 1.387, *p* < 0.05), was positive and significant, supporting and implying that the link between OCE and RQ grows stronger at higher levels of VCC. The moderating effect of VCC on the relationship between OCE and BL was negative and significant (*β* = −1.196, *t* = 2.213, *p* < 0.05); therefore, hypothesis 4, expecting a positive moderating effect, was rejected.

## Discussion

According to the findings, the shopping environment, shopping procedure, product experience, and staff service experience are all significantly correlated with relationship quality, such as brand trust, commitment, and satisfaction. According to the study, relationship quality positively correlates with brand love. As a result, hypotheses H_1_, H_2_, and H_3_ were accepted.

Hypothesis 1: This study adds to the literature by highlighting the relationship in an online setting in an e-commerce environment. Based on these findings, it can be concluded that a one-time online customer experience directly impacts a long-term phenomenon such as brand love. A better online flow state, on the other hand, aids in the development of stronger brand love because it leads to a better overall brand experience (Trivedi, 2019). The findings show that investing in a flawless online experience that immerses customers in the flow keeps them entertained and boosts brand love.

Hypothesis 2: In the long run, the online customer experience improves consumer engagement (Wirtz et al., 2012; Brodie et al., 2013). Brand marketers can engage customers in new ways using innovative digital channels and customer touchpoints like social media and mobile devices (Chan, 2012; Sashi, 2012), while customers have more opportunities to interact with their favorite brands and stay active in online brand communities (Brodie et al., 2013). This study empirically evaluated and found favorable results that improving online customer experience promoted brand love, particularly cultural framing and duration intensity (Schlobohm et al., 2016), in smartphone online shopping.

Hypothesis 3: In the new world of internet commerce, the client is no longer just the recipient of goods and services. They have evolved into participants in the creation of their value. The product in value co-creation is when the customer is enthralled and actively participates (Auh et al., 2007). Relationship quality for customer engagement and the desire to co-create in the future have been linked to improved levels of Co-creation (Frasquet-deltoro and Lorenzo-romero, 2019), increasing the number of interactions (Choo and Petrick, 2014) and brand love. Companies may use the concept of value co-creation as one of their brand love and retention strategies. According to studies, customer experience and the value co-creation process are inextricably linked. As a result, online Co-creation amplifies the impact of experiences, improving perceived value and positive attitude (Meng and Cui, 2020). The study also discovered that value co-creation has a significant moderating effect on all interactions. Except for the Delivery experience, this effect was more substantial at lower levels of value co-creation and declined as co-creation grew.

### Theoretical Implications

The OCE-BL model was created and experimentally tested in this study to look at the impact of factors that come before OCE on consumer behaviors and brand love in online shopping. Based on current literature, frameworks, and theories, the study developed an ABC and MAUT based conceptual model. Even though numerous studies have been conducted on internet businesses, few studies are available on online purchasing. An all-inclusive moderation model is also available, considering the convergence of e-commerce, customer experience, relationship quality, and new elements provided to customers by the internet, such as networking effects and value co-creation. It has not been mentioned in the current literature on online shopping.

Theoretical progress has been hampered by substituting a literature review on interpersonal love for the basic exploratory research on brand love needed to lay a solid foundation for future work. The failure to distinguish between the love emotion and the longer-lasting and more complex love relationship and the non-use of the now accepted prototype approach to identifying and defining different types of love in the brand love domain have hampered theoretical progress. As a result, this study conducted two quantitative studies (qualitative pilot study and the quantitative customer survey) using a grounded theory approach to uncover consumer-experienced features of the brand love prototype. The features were then classified as an antecedent (perceptions of high quality or qualities), the core of brand love, and the consequences of brand love in this study (brand loyalty, positive WOM, resistance to negative information, and willingness to pay a price premium). As a result, this research builds on previous work by combining constructs that had previously been studied separately and demonstrating how brand love can be used as an integrated framework for investigating how they interact. This quantitative research also shows that research on brand love derived directly from interpersonal love theories tends to overlook crucial issues such as how loved brands become a part of the consumer’s identity and provide intrinsic benefits. It is not to say that the literature on interpersonal relationships should not be used to source hypotheses or evidence supporting consumer-brand relationship research.

Second, this research has shown that the online customer experience, measured in terms of the shopping process, product experience, shopping environment, and staff services, has a significant positive effect on the relationship quality. These factors should be considered, and any barriers should be removed to motivate people to shop online. Recovery has also been critical in influencing consumer behavior and brand love in online retail. Researchers could better understand consumers’ intent and expectations in an online shopping scenario by including value co-creation as a moderator in the model. Aside from the shopping process, the numerous interactions revealed that, in the current scenario, Co-creation has a minor impact on shaping the various relationships between the antecedents and brand love for online shopping. Customers who buy things online have a higher relationship quality when co-creating value with businesses to improve their shopping experience.

Brand love is formed organically over time and results from a high-quality connection between the customer and the organization. These findings are consistent with previous research on relationship quality (satisfaction, trust, and commitment; Bowden, 2009; Hollebeek, 2011) as a mediator of brand love. Furthermore, this model backs up earlier research showing that consumer satisfaction and experience are significant determinants of brand love. However, all essential factors as antecedents and mediators were included in the model to better understand this idea in the biological route. The approach resembles relationship marketing models (Palmatier and Grewal, 2006; Aurier and Goala, 2010), in which satisfaction, trust, and commitment directly impact client buying behavior. As a component of relationship quality, only commitment directly influences engagement as a customer’s non-purchase attitude and behavior in this model (Hollebeek, 2011; Ilic et al., 2011).

Existing research has looked at elements that impact consumer satisfaction in a particular context but has not examined the link between customer experience and satisfaction in online buying scenarios. Existing research also fails to recognize the uniqueness of the circumstance in which online store business mixes and develops. The current study used value co-creation as a moderating variable to investigate changes in characteristics that impact customer satisfaction in various settings through empirical research, overcoming some of the shortcomings of prior studies. Second, given the multifaceted retail set with online store trade, the study looked at the link between customer experience and satisfaction. It systematically investigated the impact of the consuming process on customer satisfaction in an online shop setting from the perspective of customer experience, contributing to the theory of the experience mechanism of how customer satisfaction takes shape. As a result of this theoretical contribution, the link between customer experience, satisfaction, and value co-creation is integrated and expanded. Introducing new technologies, such as internet technology, has increased the rivalry in the retail business. As a result, the link between customer experience, customer satisfaction, and value co-creation has grown stronger. Integrating and using new scenarios to create exceptional customer experiences and increase customer satisfaction has become the primary road for retailers to co-create value.

From a theoretical standpoint, this research contributes to the rapidly developing literature on consumer-brand interactions by examining the function of brand love in the context of online retail firms. It is the first study in the services category to focus on an online retail brand, expanding prior research in the fields of neo-luxury, fashion, destinations, and a variety of consumer products (Whang and Florida, 2004; Ahuvia, 2005; Cho et al., 2017; Nikhashemi et al., 2019; Rodrigues and Rodrigues, 2019). Furthermore, this research focuses on the experience store format, which has never been studied in the retail research literature previously, and shows, for the first time, the relationship between the four aspects of online retail brand experiences (Brakus et al., 2009) and brand love (Bagozzi et al., 2016). This article also looks at the rise of the online retailer-as-a-brand notion, building on Burt’s (2010) research by including online customer experience characteristics. As a result, this research is vital in understanding that online customer experience is multifaceted, with varying impacts on consumer-brand interactions for each dimension. Furthermore, it was demonstrated for the first time that if the online customer experience dimensions are not properly utilized, the consumer-relationship span may be impacted in the long run. Finally, this paper adds to previous research on the evolution of positive online consumer-brand relationships (Huber et al., 2015; Rodrigues and Rodrigues, 2019) by focusing on the effects of brand relationship duration on brand love, as well as gender and educational levels, which have not previously been investigated.

The structural model’s findings reveal that, as observed in other situations, online customer experience impacts brand love (Singh et al., 2021). Regarding the demographic profile of the research participants, there are two significant contributions. Prior research has revealed that Generation Z has stronger price sensitivity (Koksal, 2019) and that millennials spend less (Moore, 2012) than older generational groups; hence this study’s excellent brand outcomes are apparent among young individuals with low-income levels (Santos and Schlesinger, 2021). This research supports the findings of Hegner et al. (2017), who claim that price has a minor effect on brand loyalty. Another discovery has to do with educational attainment. However, because the participants in this study are mostly university students, it offers a unique perspective.

Based on data from the South Asian angle, this study contributes to scholarly knowledge of online customer experience and attribution theory. To begin with, the data showed that sensory and emotional online customer experiences significantly influenced brand love in the context of the customers (Safeer et al., 2020). According to causal attributions, customers experience satisfaction and pleasure when using online businesses; they create internal feelings and affection for that brand. Consumers’ internal experiences with external forces or things (i.e., brands) lead to these attributions (Kelley, 1973). Many academics and marketers are concerned about affective and sensory brand experiences and are looking for new and novel approaches to meet customers’ requirements.

Consumer satisfaction with technology has occupied many technology studies to date. Furthermore, while marketers across industries, including banks, focus on strengthening consumer-brand connections, the impact of technology in this attempt remains understudied. According to this report, the system quality of chatbots has a more prominent part in producing a better online consumer experience. It is significant because, according to (Delone and Mclean, 2003), the weights of each quality attribute will change based on the system. It also defines the importance of online customer experience in developing brand love, a critical changing variable that indicates the strength of the consumer-brand connection. As a result, this research establishes online customer experience as a predictor of brand love.

### Practical Implications

Marketing managers should be aware of the importance of offering quality in determining consumer perceived value and the impact of these two dimensions of their perceived quality on customer satisfaction while implementing this approach. Marketing executives might use technical advancements, particularly in a social media brand community, to improve the quality of customer relationships and the firm’s value offer. For example, the online brand community offers a variety of unique perks that help online clients have a better experience with the company’s products and services (Gummerus et al., 2012; Wirtz et al., 2012). On the other hand, perceived quality and value are insufficient for experience development, requiring improving relationship quality. Previous research in relationship marketing (Palmatier and Grewal, 2006; Aurier and Goala, 2010) and online relationship marketing (Verma et al., 2015; Steinhoff et al., 2019) suggest creating and sustaining client connections in this respect.

Companies must manage online retail stores to boost customer experience and relationship quality. It can assist online merchants in gaining a better and more rapid grasp of the online customer experience and brand love goals. Online retailers should: (1) diversify their promotional methods, attracting customers through multiple channels and establishing a solid relationship with them through online marketing on mobile devices; (2) generate traffic through social media and promotional coupons for customers and followers; and (3) use affiliate marketing to reach target customer groups. They should establish a scientific and practical sales management system, consider the impact of gender on the relationship quality between online customer experience and brand love, provide the best shopping experience for customers, build a good brand image, and launch customer-centric marketing campaigns in order to achieve a sustainable competitive differentiation. Finally, retailers should connect online channels to provide consumers with the ideal shopping experience: (1) they should use digital technology to create two-way interconnectivity; (2) they should connect the coupling channel of online store commerce and establish a complete system network; (3) they should rely on system synergy and data analysis to facilitate the optimization of online customer experience and effectively better sustain long-term relationship quality.

The findings revealed that the delivery experience during the shopping procedure was critical in generating brand love in online shopping. Customers are concerned about finding out where their things are and when they will be delivered. Customers save time and avoid the stress of standing in lengthy lines thanks to the product’s ease and doorstep delivery. Customers want to be a part of the co-creation process to create value for themselves through an efficient delivery experience. It might be because the consumer has already committed time, energy, and, in some cases, money to purchase the items and attempts to become more active in the co-creation process to guarantee that delivery is efficient. As a result, it is proposed that retailers aim to include customers in the delivery process.

Other criteria that did not directly impact the relationship quality in online purchasing were privacy and security in Product experience. However, in the presence of Co-creation as a moderator, these characteristics affected consumers’ desire to repurchase considerably, albeit at a lesser degree of involvement. Improved privacy and security features should be used by online businesses to continue influencing customers’ attitudes. They should utilize encryption software and anti-hacking technology to protect clients’ information such as credit card numbers, phone numbers, addresses, and account numbers. Customers prefer online payments in this era of an epidemic because it is a safer alternative, but e-retailers should strive to protect them.

Our results suggest that managers better tune their marketing techniques in inactivity, content, and communication. Furthermore, it would be fascinating to see if community members disseminate WOM merely because of their trust and commitment to the brand fan page and the strength of their brand connection. Brand managers use Facebook and Instagram to establish better relationships beyond the purchase. In light of these findings, practitioners should strengthen their customer relationship management (CRM) efforts by leveraging their Facebook and Instagram fan pages to send marketing messages to their target consumers with particular activities. Because communities tend to be less mercantilist and more immediate, they frequently become a preferred communication channel between the business and the customers. Using a brand fan page to increase brand relationship quality is valuable.

Third, economic advantages related to the consumer’s ongoing relationship with the brand might include incentives for involvement or time savings through preferential treatment that encourages quicker selections. On the other hand, providing economic benefits should be combined with a strategy that provides supporters with amusement. Firms should design webpage features and functions of their brand fan pages to deliver more hedonic benefits better; that is, they should, for example, offer daily content with fun and entertaining characters, such as videos, photos, texts, games, and Facebook Live (a live streaming video channel to keep users updated on what is happening on Facebook with positive information about the brand). It lets you turn your brand into a trust, commitment, and satisfaction center for long-term brand relationship quality.

According to the findings, marketers should prioritize the online sensory and affective experience above the intellectual and behavioral ones. The design, aesthetic impressions, and usages contribute to the sensory experience (Brakus et al., 2009). Marketers may tweak their services to include attractive graphics, typography, a well-balanced color palette, and high-definition imagery. They can also concentrate on creating appealing sound effects and a pleasant-to-use controller. Managers can transmit customers’ feelings when using the service to improve the emotional experience during online purchases. Professionals can stress the construction of intriguing information, compelling narrative, and appealing slogans for the intellectual experience. They can also allow customers to rate the material or use an algorithm tailored to their preferences.

Marketers can be aware of customer habits while utilizing the service, such as checking their phones or having appetizers, for the behavioral experience. Furthermore, to create and maintain an emotional bond with customers, brand managers should include the essence of the service in their marketing strategies, which aims to satisfy the need for escapism and hedonism through brand identification, self-image, and enhancement of positive emotions. According to the findings of this study, brand managers may consider youth an appealing market. Brand managers using this technique will boost customer brand love while improving financial performance. To accomplish critical brand goals, brand managers should concentrate their efforts on the online customer experience and brand love.

This research seeks to explain the notion of value co-creation from a managerial viewpoint, which can assist managers in acquiring a thorough awareness of the intricacies of this concept before applying it as a strategic intervention. That is, this research will assist managers in comprehending the essential conception of value co-creation as a strategy, allowing them to view this notion as a strategic intervention for their companies. Furthermore, this research will assist managers in finding sources that might give them valuable practical insights into this notion.

According to this study, brand love was favorably affected by intellectual, behavioral, emotional, and sensory online encounters. To improve the quality of their connection in customers’ eyes, global managers should establish brands that meet their promises *via* quality commitment, honesty, and reflection of legacy. Asians follow Western trends and are eager to buy global brands (Joachim-Fuchs, 2003). As a result, pleasant multimodal online experiences aid managers in retaining existing clients and gaining new ones in Pakistan. These findings revealed that the quality of a connection has a significant impact on brand loyalty.

Finally, this study demonstrated the mediation impact of relationship quality in the multidimensional online customer experience and brand love connections. Companies are recommended to use experiential marketing to establish and sustain credibility in global brands by implementing various customer connection methods to develop brand-loving customers (Safeer et al., 2020). Alternatively, corporations should position their brands through sensory and intellectual marketing techniques by enhancing relationship quality to please customers intellectually. These methods may be used *via* social media platforms such as Instagram, Facebook, and Twitter to improve consumer views of businesses and boost customer equity (Yu and Yuan, 2019). Managers should supply tailored services for a practical and behavioral online client experience. These techniques can improve the quality of relationships between global brands and firms, resulting in good customer feedback and the development of brand love.

### Limitations and Future Research Directions

This work has some limitations, and researchers may revisit it in the future. Although the sample consisted of most students representing different social strata from different cities, the online customers other than students were minimal. It may inhibit the generalizability of findings to all online customers. As a result, to generalize the findings, the study needs to be replicated on a larger sample size in diverse and online consumers in other countries. The study was primarily a cross-sectional, one-time survey focused on cost and time constraints.

On the other hand, a long-term longitudinal study would contribute more to the field of knowledge. This research focused on business-to-consumer (B2C) online commerce and may not apply to other types of online businesses, such as B2B or others. More research into these aspects and other new online customer experiences could examine various behavioral intents and outcomes. Even though both positive and negative factors influence relationship quality, previous research has primarily focused on it as a positive term.

Furthermore, this model suffers from customer-related issues because previous research has primarily focused on firm antecedents and consequences of brand love. Customers’ reactions to advertised engagement activities and the formation of customer relationships, for example, will be influenced by psychological mediators. Similarly, the result simply reflects the firm’s benefits of relationship quality because it lacks customer-related outcomes.

## Conclusion

As a result of the COVID19 epidemic, e-commerce adoption rates increased due to the increased use of online media. During these challenging times for e-businesses, providing flawless customer experiences and forging long-term, profitable relationships with clients have become more critical. The findings of this study show that a positive online customer experience has an indirect positive impact on brand love through improved relationship quality. Therefore, online shopping should give customers a fully immersive and high-quality experience with a brand’s website.

As a result, it is unlikely to affect long-term phenomena like brand love. Although online shopping has been studied from various angles, the number of studies examining the impact of online shopping on the overall online customer experience and brand love is limited in the current literature. However, that study only looked at the links between online customer experience and brand love. This study used contemporary methodology and data analysis techniques to add to existing research by introducing the brand love dimension and evaluating the combined direct and indirect effects of those constructs’ phenomena.

According to the findings, encouraging a flow state should not be considered a nice-to-have feature in an e-commerce scenario. However, doing so helps e-commerce businesses achieve their long-term objectives. When it comes to brand love, the time customers spend on an online platform for a product, the amount of time they spend there, and the pleasure they derive are critical factors. In enabling and making the shopping process enjoyable, customized and individualized products and influencing aesthetic designs on online platforms produce a more crucial online customer experience than the purchase experience. Finally, websites that keep customers engaged will be preferred.

Finally, this research aims to add to previous work on customer emotions and affections in online shopping and love. The study investigated retailing on an online platform using the MAUT and the ABC theory, focusing on value co-creation. Customers and retailers collaborate to develop products and services. The antecedents of online customer experience influencing customers’ affection for online shopping were examined. The other purpose of this study was to see if value co-creation had any moderating effects on the relationship between online customer experience antecedents and relationship quality with online brands. The results supported the research hypotheses in light of value co-creation. According to the findings, all linkages have strong value co-creation moderation effects.

## Data Availability Statement

The raw data supporting the conclusions of this article will be made available by the authors, without undue reservation.

## Ethics Statement

Ethical review and approval was not required for the study on human participants in accordance with the local legislation and institutional requirements. The participants provided their online informed consent to participate in this survey.

## Author Contributions

All authors listed have made a substantial, direct, and intellectual contribution to the work and approved it for publication.

## Funding

The article processing cost (APC) was partially financed through the publication incentive fund 2022 by the Universidad Andrés Bello (Code: C.C. 21500) and the Universidad Autónoma de Chile (Code: C.C. 456001).

## Conflict of Interest

The authors declare that the research was conducted without any commercial or financial relationships that could be construed as a potential conflict of interest.

## Publisher’s Note

All claims expressed in this article are solely those of the authors and do not necessarily represent those of their affiliated organizations, or those of the publisher, the editors and the reviewers. Any product that may be evaluated in this article, or claim that may be made by its manufacturer, is not guaranteed or endorsed by the publisher.
